# Analysis of risk factors for perioperative death in patients undergoing aortic valve replacement using biological valves

**DOI:** 10.1097/MD.0000000000023909

**Published:** 2020-12-24

**Authors:** Qi Li, Hongbo Gao, Qiuxia Ji, Jianshu Song, Longfei Li, Xu Liu

**Affiliations:** a Department of Medicine, Qingdao University; b Department of Cardiovascular Surgery, Affiliated Hospital of Qingdao University, Qingdao, China.

**Keywords:** aortic valve disease, aortic valve replacement, biological valve, perioperative mortality

## Abstract

**Background:**

Aortic valve disease has become one of the important factors affecting human health. Aortic valve disease is a progressive disease, if not actively treated, the prognosis is poor. Aortic valve replacement (AVR) surgery is an important treatment for aortic valve disease. At present, the AVR surgery using biological valve accounts for about 40% of the total number of AVR surgery. There are still more perioperative deaths in China due to the large number of AVR patients using biological valves. The objective of this study is to explore measures to reduce perioperative mortality of patients after AVR surgery with biological valves.

**Methods:**

The clinical data of patients undergoing AVR surgery with biological valves in Affiliated Hospital of Qingdao University from November 15, 2020 to December 31, 2022 were reviewed and analyzed. Patients were divided into death group and survival group according to their perioperative survival. Risk factors that may influence perioperative mortality were analyzed and compared between the 2 groups.

**Discussion:**

This study was a retrospective analysis of risk factors that may influence perioperative mortality in patients undergoing AVR surgery using biological valves. The conclusions of this study can be used to guide clinical decisions-making and relevant guidelines-developing for perioperative treatment of patients undergoing AVR surgery using biological valves.

## 1 Introduction

In recent years, the incidence of aortic valve disease has been on the rise.^[1]^ Aortic valve disease has become one of the important factors affecting human health.^[2]^ Aortic valve disease is a progressive disease, if not actively treated, the prognosis is poor. Medical treatment is feasible for patients with mild lesions. When patients have severe lesions or obvious symptoms, aortic valve replacement (AVR) surgery is required to treat them.^[3]^ In AVR surgery, there are mainly 2 kinds of artificial valve: mechanical valve and biological valve.^[4]^

Mechanical valve has been used in clinic since 1950s and is still the main replacement valve. However, patients with mechanical aortic valve replacement need to take anticoagulant drugs for life, and there is a high risk of complications such as thromboembolism and bleeding. Biological valve has been used in clinical practice since the 1960s. Although patients who use biological valve for AVR avoid life-long anticoagulants, they face problems such as structural valve decay. At present, the AVR surgery using biological valve accounts for about 40% of the total number of AVR surgery.^[5,6]^

It was found that there was no difference in systemic embolism, valve thrombosis, infective endocarditis with artificial valves, valvular complications, and long-term survival between biological and mechanical valves at 15 to 20 years of long-term follow-up, but the risk of bleeding with mechanical valves was higher. However, there was a higher rate of biological valve decay in patients younger than 65 years of age, and this risk of biological valve decay was more pronounced in AVR surgery.^[7]^

There are still more perioperative deaths in China due to the large number of AVR patients using biological valves.^[8,9]^ Therefore, we conducted this retrospective study. By analyzing the risk factors that may affect the perioperative mortality of patients undergoing AVR surgery using biological valves (we defined the period from admission to postoperative discharge as the perioperative period of patients), we want to improve the postoperative survival rate of patients undergoing AVR surgery with biological valves and to provide evidence-based medical reference for clinical work.

## 2 Objective

By analyzing the risk factors for perioperative mortality of patients after AVR surgery with biological valves, we explored measures to reduce perioperative mortality of patients after AVR surgery with biological valves. Conclusions of our study will help clinicians improve the perioperative survival of patients undergoing AVR surgery using biological valves.

## 3 Methods

### 3.1 Study design and setting

The clinical data of patients undergoing AVR surgery with biological valves in Affiliated Hospital of Qingdao University from November 15, 2020 to December 31, 2022 were reviewed and analyzed. Patients were divided into death group and survival group according to their perioperative survival. QL and LL will generate the allocation sequence, HG and QJ will enrol participants. Risk factors that may influence perioperative mortality were analyzed and compared between the 2 groups. The study has been approved by the Medical Ethics Committee of the Affiliated Hospital of Qingdao University (QYFYWZLL25536). And this study was registered in the Chinese Clinical Trial Registry (ChiCTR2000040097) November 21, 2020.

The protocol conforms to the 2013 SPIRIT (standard protocol items: Recommendations for Interventional Trials) statement.

### 3.2 Participants

#### 3.2.1 Inclusion criteria

Aortic valve disease was diagnosed by cardiac ultrasound. In line with the indications of aortic valve replacement surgery. The patients have no life-threatening major diseases. Patients undergoing AVR surgery using biological valves. The patients agreed to participate in this study and signed the informed consents.

#### 3.2.2 Exclusion criteria

Patients with incomplete clinical data. In addition to heart disease, patients with end-stage diseases of various organs or patients with other life-threatening diseases. Patients transferred to or discharged from hospital due to personal reasons. Patients who lost contact during follow-up. The patients did not use biological valves for AVR surgery.

### 3.3 Outcome measures

#### 3.3.1 Primary outcome

The clinical data of patients were collected independently by 2 researchers. The main evaluation and analysis outcome measures collected in this study included preoperative New York Heart Association cardiac function classification, operation time, surgical approach (transcatheter AVR or surgical AVR), aortic occlusion time, ventilator assisted time, patients’ extracorporeal circulation time, postoperative hospitalization days, postoperative complications such as arrhythmia, perivalve leakage, atrial thrombosis and so on, and whether the patients received secondary surgery during hospitalization.

#### 3.3.2 Secondary outcomes

The secondary evaluation and analysis indicators collected in this study included the basic data of patients who underwent AVR surgery using biological valves, including sex, age, height, weight, length of disease, and other diseases other than aortic valve lesions. Data on secondary indicators were also collected independently by the 2 researchers.

### 3.4 Blinding

Because this study is a retrospective study, it is impossible to be blind in data collection and grouping. Therefore, we are blind to analysts in data analysis.

### 3.5 Sample size estimation

According to the literature, the mortality rate of patients undergoing AVR surgery using biological valves was 11.7%.^[10]^ In this study, we preliminarily plan to collect relevant data of 110 patients who underwent AVR surgery using biological valves, including 20 patients in the death group and 90 patients in the survival group. Because this study is a retrospective study, the loss of follow-up of patients is not considered.

### 3.6 Data management

All data collected in this study will be recorded in the case record forms (CRFs), which will be entered into the database of our study by 2 independent people and cross-checked. When data collection is complete, the database will be analyzed by professional medical statisticians. Data from patients participating in this study will be protected and used for this study only. The personal information of the participants will be kept strictly confidential.

### 3.7 Statistical analysis

Symmetric continuous variables will use the mean and standard deviation, while asymmetric continuous variables will use the median. Use frequency and percentage to describe categorical variables. In the intergroup analysis, variance analysis was used for continuous variables and chi-square test was used for classified variables. We will also use univariate logistic regression analysis and multivariate logistic regression analysis to explore the relationship between risk factors and perioperative death in patients with AVR surgery using biological valves. We will use R software (https://www.r-project.org/) and Empower(R) to analyze the data statistically, and set *P* < .05 as the statistically significant.

## 4 Discussion

This study was a retrospective analysis of risk factors that may influence perioperative mortality in patients undergoing AVR surgery using biological valves. Our previous plan was to collect data from 110 patients and divide them into the death group and the survival group for comparative analysis. In practice, we collect as many patient data as possible that meet the inclusion criteria. Try to find out as accurately as possible the relevant risk factors affecting the perioperative mortality of patients undergoing AVR surgery with biological valves, and to intervene these risk factors early in clinical work. Reduce perioperative mortality in patients undergoing AVR surgery with biological valves to reduce the perioperative mortality of patients undergoing AVR surgery using biological valves.

The conclusions of this study can be used to guide clinical decisions-making and relevant guidelines-developing for perioperative treatment of patients undergoing AVR surgery using biological valves.

## Author contributions

**Conceptualization:** Qi Li, Xu Liu.

**Data curation:** Qi Li, Qiuxia Ji.

**Formal analysis:** Qiuxia Ji, Jianshu Song, Xu Liu.

**Funding acquisition:** Xu Liu.

**Investigation:** Qi Li, Jianshu Song.

**Methodology:** Qi Li, Hongbo Gao, Qiuxia Ji.

**Project administration:** Hongbo Gao, Jianshu Song.

**Software:** Hongbo Gao, Longfei Li.

**Supervision:** Xu Liu.

**Writing – original draft:** Qi Li, Hongbo Gao, Longfei Li.

**Writing – review & editing:** Qiuxia Ji, Longfei Li.
